# Subzero Nonfreezing Hypothermia with Insect Antifreeze Protein Dramatically Improves Survival Rate of Mammalian Cells

**DOI:** 10.3390/ijms222312680

**Published:** 2021-11-24

**Authors:** Akari Yamauchi, Ai Miura, Hidemasa Kondo, Tatsuya Arai, Yuji C. Sasaki, Sakae Tsuda

**Affiliations:** 1 Graduate School of Life Sciences, Hokkaido University, Sapporo 060-0810, Japan; akari.yamauchi.jp@gmail.com (A.Y.); h.kondo@aist.go.jp (H.K.); 2 Bioproduction Research Institute, National Institute of Advanced Industrial Science and Technology (AIST), Sapporo 062-8517, Japan; a.miura@aist.go.jp; 3 Graduate School of Frontier Sciences, The University of Tokyo, Kashiwa 277-8561, Japan; t.arai@edu.k.u-tokyo.ac.jp (T.A.); ycsasaki@edu.k.u-tokyo.ac.jp (Y.C.S.); 4 OPERANDO Open Innovation Laboratory, National Institute of Advanced Industrial Science and Technology (AIST), Tsukuba 305-8563, Japan

**Keywords:** cell preservation, supercooling, antifreeze protein (AFP), Ice binding, membrane protection, ice-like clathrate waters

## Abstract

Cells for therapeutic use are often preserved at +4 °C, and the storage period is generally limited to 2–3 days. Here, we report that the survival rate (%) of mammalian cells is improved to 10–20 days when they are preserved with a subzero supercooled solution containing the antifreeze protein (AFP), for which an ability to stabilize both supercooled water and cell membrane integrity has been postulated. We chose adherent rat insulinoma (RIN-5F) cells as the preservation target, which were immersed into −5 °C-, −2 °C-, or +4 °C-chilled “unfrozen” solution of Euro-Collins or University of Washington (UW) containing the AFP sample obtained from insect or fish. Our results show that the survival rate of the cells preserved with the solution containing insect AFP was always higher than that of the fish AFP solution. A combination of the −5 °C-supercooling and insect AFP gave the best preservation result, namely, UW solution containing insect AFP kept 53% of the cells alive, even after 20 days of preservation at −5 °C. The insect AFP locates highly organized ice-like waters on its molecular surface. Such waters may bind to semiclathrate waters constructing both embryonic ice crystals and a membrane–water interface in the supercooled solution, thereby protecting the cells from damage due to chilling.

## 1. Introduction

A body temperature below 35 °C is described as hypothermia, which is sometimes used as a medical protocol, since lowering body temperature to 4−35 °C reduces the metabolic rate and minimizes the energy consumption of cells, thereby improving their viability [1]. For this reason, hypothermic methods are utilized for transplantation, short-term storage, medical shipping, and livestock farming of cells, tissues, and organs [1]. For example, human liver is generally preserved with a +4 °C hypothermic protocol, which can extend its lifetime up to a maximum of 12 h [1]. In contrast, less is known about the effects of applying temperatures below 0 °C. Such a physically metastable (unfrozen) water state is called supercooling, in which numerous H_3_O+ molecules are assumed to form tiny embryonic ice crystals. Once they cluster and take on a marginal size, the supercooling loses its equilibrium, and the water transforms into a multicrystalline state, leading to the formation of ice [2,3,4]. Ice physically destroys the structure of cells; therefore, the inhibition of embryonic ice crystal growth below 0 °C is desirable to preserve the cells. A recent study reported that a subzero nonfreezing hypothermia with University of Wisconsin (UW) solution chilled to −4 °C can prolong the lifespan of human liver to 27 h [5]. Biochemical processes, such as metabolism, decrease by at least half with each 10 °C temperature drop. As such, tissues may undergo extremely low levels, i.e., essentially zero, of endogenous metabolic activity at around −5 °C compared with hypothermic storage at +4 °C [6]. Hence, the preservation of certain cells through subzero nonfreezing hypothermia may ensure the viability of those cells for a much longer period than previously thought.

One question is how to minimize supercooling-induced damage to the cells, since subzero temperatures severely affect the ionic regulation across the membrane, as well as the oxidation of proteins and phospholipids, compared with the ordinary +4 °C preservation (Figure S1) [1,7,8,9,10,11]. Indeed, it has been shown that a temperature change from 37 °C to hypothermia causes lipid phase transition from liquid crystal to the gel state, resulting in a loss of membrane integrity [9]. This causes osmotic imbalance in- and outside of the cell, which leads to irreversible swelling and rupture of cell wall. Therefore, maintaining membrane integrity should be the primary requirement for hypothermic cell preservation.

Antifreeze proteins (AFPs) purified from cold-adapted organisms are extraordinary macromolecules that commonly bind to embryonic ice crystals to inhibit their growth [12,13,14], and to the cell membrane to improve its integrity [15,16]. Fish-derived AFPs are categorized as type I–III and antifreeze glycoprotein (AFGP). AFP I is an alanine-rich amphipathic α-helical polypeptide (*M*_w_ = 3.5 kDa); AFP II is a globular protein with significant structural similarity with the C-type lectin (*M*_w_ = 14 kDa); AFP III is another globular protein composed of twisted loops folded into triple-strand β-sheets (*M*_w_ = 6.5 kDa); and AFGP (*M*_w_ = 2.6–34 kDa) is a repetitive polypeptide of (Ala–Ala–Thr)_n_ (*n* = 4–50), in which C_β_ of Thr is glycosylated with a disaccharide β-D-galactosyl-(1 3)-α-N-acetyl-D-galactosamine [12]. A β-helical AFP (*M*_w_ = 9 kDa) was discovered from the larvae of the beetle *Tenebrio molitor* (denoted *Tm*AFP) [12]. Fish and insect AFPs have been shown to locate ice-like organized waters on their surfaces, which are thought to undergo complementary binding to the waters comprising the basal, prism, and/or pyramidal planes of single ice crystals [17,18]. The potential for specific or multiple ice plane binding depends on the AFP type. The ice-binding of AFP modifies a single ice crystal into a bipyramid or a lemon-like morphology, according to the ice plane specificity [19,20]. Ice-binding also depresses the freezing point (*T*_f_) of the solution without significantly affecting the melting point (*T*_m_), where the temperature difference between *T*_m_ and *T*_f_ is called thermal hysteresis (TH) [21,22]. AFPs are categorized into hyperactive or moderately active species according to their TH activity [19,23,24].

The membrane-protection ability of AFPs prolongs the lifespan of mammalian cells by inhibiting swelling and rupturing [15,16,25,26]. This characteristic was first reported for the fish-derived AFGP in 1990 [15]. AFGP and AFP I–III were found to maintain the integrity of pig oocyte oolemma by preventing ion leakage across the membrane at +4 °C, which inhibits the rupturing of preserved cells [25,27,28,29]. Kamijima et al. reported that 10 mg/mL of the fish AFP I and III could keep 60% of rat insulinoma (RIN-5F) cells alive after 5 days of preservation at +4 °C [26]. The preserved cells maintained the ability to secrete insulin. More data have demonstrated that both AFGP and AFP I–III prolong the lifespans of cells, tissues, and organs at +4 °C [15,16,30,31,32,33]. It should be noted that the cell-preservation function of AFPs requires the coexistence of low molecular weight solutes, such as polyhydroxy alcohol (ex. glycerol), sugars (ex. trehalose), amino acids (ex. proline), and/or glycolipids [34]. This notwithstanding, the question of whether AFPs improve the viability of preserved cells at subzero temperatures remains open. Here, we examined the survival rate (%) of RIN-5F cells after 1, 3, 5, 10 and 20 days of “nonfreezing” preservation at temperatures of −5 °C, −2 °C, or +4 °C by employing commercial cell storage solutions, in which we dissolved fish- or insect-derived AFP. A potential mechanism for the significant improvement in viability detected with insect AFP (*Tm*AFP) is discussed based on its tertiary structure that locates regularly arrayed ice-like waters.

## 2. Results and Discussion

### 2.1. Activity of AFPs in the Cell-Preservation Solution

**Figure 1:**
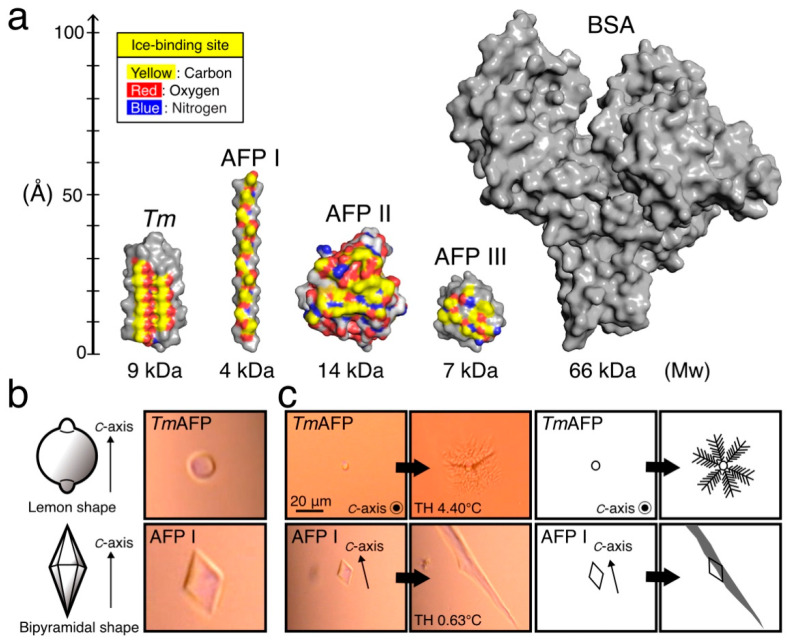
Protein samples used for the present cell preservation experiments. (**a**). Structure of antifreeze proteins (AFPs) and bovine serum albumin (BSA), for which surface model was prepared with Pymol (https://pymol.org/2/ Accessed date: 24 November 2021). *Tm* denotes *Tm*AFP from the *Tenebrio molitor* beetle (PDB code = 1EZG) [35]. AFP I from barfin plaice is assumed to form an alpha-helix, similarly to AFP I from winter flounder (1WFB) [36]. AFP II from longsnout poacher creates an elongated globular structure (2ZIB) [37]. AFP III from notched-fin eelpout creates more compact globules (5XQN) [38]. BSA (3V03) is 5–16 times larger than AFPs [39]. The C-, O-, and N-atoms constructing the ice-binding sites on each AFP molecule are indicated with yellow, red, and blue, respectively. (**b**). Illustrations and actual photomicroscope images of a single ice crystal observed for 1.5 mM AFP-dissolved EC solution. The crystal forms a lemon-like morphology when it dissolves *Tm*AFP, while it changes into a bipyramid by dissolving AFP I. (**c**). Bursting crystal growth observed for a single ice crystal created in EC solution containing 1.5 mM AFP with illustrated interpretations. In the *Tm*AFP solution, the crystal bursts in six directions on the *c*-axis (basal plane), creating a dendritic pattern. For the AFP I solution, the bursting progresses along the *c*-axis to create a needle-like pattern. The TH values were 4.40 °C and 0.63 °C in the *Tm*AFP- and AFP I-dissolved EC solution, respectively.

Figure 1a compares tertiary structures of the AFP samples used in the present nonfreezing hypothermic cell preservation experiments, where only one representative isoform among the 7–13 isoforms identified for each AFP species is indicated. The structures include the bovine serum albumin (BSA) used as a control. As shown, *Tm*AFP is a relatively thick, pole-shaped molecule [35]. AFP I constructs a thinner pole comprising an amphiphilic α-helical peptide [36]. AFP II forms a cysteine-rich globular protein made of several α-helices and β-sheets [37]. AFP III is a smaller globular protein composed of many β-sheets [38]. Finally, BSA is a 67 kDa huge globular protein [39], for which cell protection ability has been suggested.

The yellow-colored region of each AFP species indicates the ice-binding site (IBS). *Tm*AFP notably locates linearly aligned oxygen atoms with regular intervals in IBS, as illustrated by their rib-like pattern colored yellow and red (Figure 1a, left). It is speculated that these oxygen atoms undergo complementary binding to the latticed water atoms, thereby constructing a single ice crystal [13,18]. For the other AFPs, polar atoms including oxygens located on their surface also show position matches to the water constructing an ice crystal surface. Note that polar atoms are immobilized by their neighboring hydrophobic residues in IBS [12,38]. Overall, the oxygen atoms located on an AFP molecule are organized into an ice-like arrangement with which AFP binds to an embryo ice crystal. The linearly aligned oxygen atoms on *Tm*AFP were thought to bind more perfectly to the latticed waters, leading to the *Tm*AFP-binding of an embryonic ice crystal [22].

To confirm the functional ability of the aforementioned AFPs, we examined the morphology of a single ice crystal and the TH value for each sample dissolved in the EC solution. Note that the unit structure of a single ice crystal at one atom is a hexagonal cylinder, which is defined by three equivalent *a*-axes (*a*_1_–*a*_3_) perpendicular to the *c*-axis [17,40]. The two top planes in the hexagonal shape are called basal planes. The *c*-axis penetrates the middle of the basal planes of the hexagonal cylinder from bottom to top. The AFP-binding to the basal plane is known to be a key determinant for hyperactivity [19,35]. As shown in Figure 1b, a rounded lemon-like ice crystal morphology was observed for *Tm*AFP (Figure 1b), while the ice crystal exhibited a hexagonal bipyramid for AFP I. The former is ascribed to the binding ability of hyperactive AFP to multiple ice planes including the basal plane, and the latter to the binding of moderately active AFP to only specific ice planes [12,19]. The lemon-like crystal of *Tm*AFP underwent bursting growth in six directions, creating a vein-like pattern, while AFP I exhibited needle-like ice growth (Figure 1c). These observations are also attributable to multiple ice-plane-binding of *Tm*AFP and specific ice-plane-binding of AFP I, respectively. AFP I-type changes were also observed for AFP II and III. The TH values were 4.4 °C for 1.5 mM of *Tm*AFP solution and approximately 1 °C for AFP I–III samples, which are typical values for each AFP, suggesting that *Tm*AFP functions as a hyperactive AFP species and fish AFP I–III as moderately active species [19], even in the EC solution. The present AFP samples are therefore thought to have maintained their original structure and function throughout the cell preservation experiments.

### 2.2. Preliminary Cell-Preservation Tests with TmAFP

**Figure 2:**
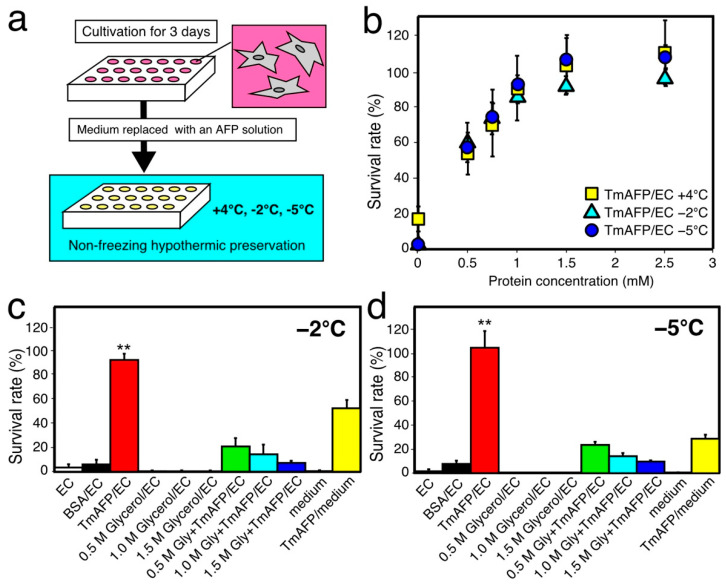
Survival rate of RIN-5F cells after 1 day of hypothermic preservation. (**a**). A flow chart of the preservation experiment. The cells were cultivated for 3 days at 37 °C in a 96-well plate before preservation experiments. (**b**). Survival rate (%) of RIN-5F cells evaluated at +4 °C, −2 °C, and −5 °C with Euro Colins (EC) solution containing 0–2.5 mM of *Tm*AFP. (**c**,**d**). The survival rates evaluated at −2 °C and −5 °C with the EC solution containing 0–1.5 M of glycerol and/or 1.5 mM *Tm*AFP. Asterisk shows that the *p*-value vs. control (EC solution) is statistically significant (*p* < 0.01).

For the initial hypothermic cell-preservation test, we examined the survival rate (%) of rat insulinoma (RIN-5F) cells by using the native *Tm*AFP sample for a 1-day period (Figure 2). When the RIN-5F cells reached 80% confluent state with the RPMI-1640 medium after 3 days of incubation at 37 °C, the medium was replaced with EC solution or solution containing *Tm*AFP to start the 1-day preservation experiment at −5, −2, and +4 °C (Figure 2b). At +4 °C, a 17% survival rate was obtained with only EC solution without AFP. However, this solution could not maintain cell viability (~0%) at −2 °C and −5 °C for 1 day by itself. In contrast, the *Tm*AFP-dissolved EC solution (Figure 2b) could maintain cell viability at −5 °C, −2 °C, and +4 °C. To evaluate the optimal *Tm*AFP concentration for cell preservation, we examined the cell survival rate (%) in a range of concentrations between 0.5 and 2.5 mM. The survival rate exhibited a hyperbolic dependence on *Tm*AFP concentration (Figure 2b), similarly to the TH dependence of an AFP species [19,23]. The hyperbolic profile shows that the survival rate reached approximately 100% when the *Tm*AFP concentration reached 1.5 mM for all three temperatures. We therefore chose 1.5 mM as the standard concentration for all AFP samples in order to compare their preservation ability with RIN-5F cells. Tomalty, H.E. et al. (2019) reported that 100 µg/mL *Tm*AFP exhibited no cytotoxicity on HEK 293 T cells, even after 3 days of incubation [41]. The present results verify the nontoxicity of *Tm*AFP, and, for the first time, demonstrate its cell-protection ability for mammalian cells.

Glycerol generally functions as a superior cryoprotectant, improving cell viability under supercooled conditions [34]. This compound is also known to be synthesized in insect body, and condensed in the hemolymph to prevent freezing at concentrations of 0.5–1.5 M during the winter. We therefore examined the protection effects of such amounts of glycerol on RIN-5F cells after 1 day of preservation at −2 °C and −5 °C. The cells preserved with EC solution containing 0.5, 1.0, and 1.5 M of glycerol did not survive at either temperature (Figure 2c,d and Figure S3), i.e., glycerol did not improve RIN-5F viability. In contrast, a 92% survival rate was observed when cells were preserved at −2 °C for 1 day with EC solution containing 1.5 mM *Tm*AFP (Figure 2b,c). At −5 °C, the cell survival rate with *Tm*AFP improved to approximately 100% (Figure 2b,d). The cells did not survive 1 day of preservation at −2 °C and −5 °C with the EC-solution containing BSA, as it has no ice-binding ability. The cells did not survive with the EC solution containing 0.5–1.5 M of glycerol, while their survival rate (%) became appreciable when the solution contained 1.5 mM of *Tm*AFP at both −2 °C and −5 °C (Figure 2b,d). These results suggest that *Tm*AFP positively contributes to RIN-5F viability under supercool temperatures. This is in good agreement with previous indications that AFP plays a significant role in protecting the cells and tissues from freezing in cold-tolerant organisms in nature [2,18,34]. 

**Figure 3:**
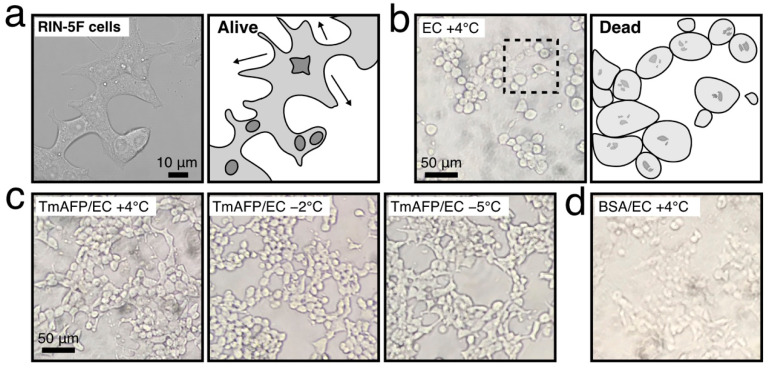
Photomicroscope image of RIN-5F cells before and after 1 day of hypothermic preservation. (**a**). Image of the living cells, captured before preservation. The cells are mostly clumped together and elongated. (**b**). Image of dead cells. An illustrated interpretation is depicted for the hatched square. The cells are distinctly rounded and collapsed. (**c**). Images of living cells after nonfreezing hypothermic preservation with EC solution containing 1.5 mM of *Tm*AFP at +4 °C, −2 °C, and −5 °C. (**d**). Cells preserved with EC solution containing 1.5 mM of BSA at +4 °C. The cells are mostly rounded and not clumped together.

Photomicroscope images of cells are also informative regarding their viability (Figure 3). Namely, living cells observed in the cultivation medium are clumped together and elongated around one another (Figure 3a). In contrast, dead cells are distinctly rounded and detached (Figure 3b). The swelling of cells was also detected during the +4 °C preservation and in the present experiments; this was ascribed to chilling damage [25,26]. As shown in Figure 3c, the RIN-5F cells were clumped together and elongated, as in Figure 3a, during nonfreezing hypothermic preservation at +4 °C-, −2 °C, and −5 °C with *Tm*AFP, suggesting a superior cell-protection ability of this protein. In contrast, the cells preserved with the BSA-containing EC solution were rounded and detached (Figure 3d), suggesting the ineffectiveness of BSA. Note that the cells were mostly rounded and ruptured when preserved with glycerol-dissolved EC solution (Figure S3), while they tended to be clumped together when *Tm*AFP was added to the solution. These results suggest that *Tm*AFP binds to the lipid bilayer of RIN-5F cells to stabilize their membrane integrity, similarly to fish AFPs [25].

### 2.3. Comparison of Cell-Preservation Ability between the AFP Species

**Figure 4:**
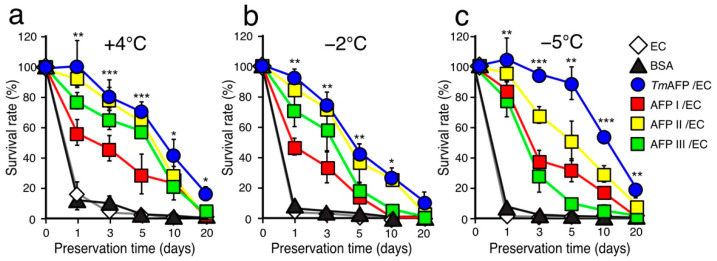
Survival rate (%) of RIN-5F cells preserved with EC solution containing AFP. (**a**). Time-dependence of the cell survival rate (%) after nonfreezing hypothermic preservation with the EC solution containing 1.5 mM of BSA, *Tm*AFP, or AFPI–III at +4 °C. (**b**). Time-dependence data obtained at −2 °C. (**c**). Time-dependence data obtained at −5 °C. The solutions were not frozen at −2 °C and −5 °C during the preservation experiments for a maximum of 20 days. The *Tm*AFP-containing EC solution always gave the best results (blue circles). Asterisk shows that *p*-value vs. control (EC solution) is statistically significant (* *p* < 0.05, ** *p* < 0.01, *** *p* < 0.001).

A +4 °C-hypothermic cell preservation ability for 24–96 h has been reported for fish AFP I–III [16,26]. Here, we examined the survival rate (%) of RIN-5F cells between AFP I–III and *Tm*AFP after 1, 3, 5, 10 and 20 days of preservation at +4 °C, −2 °C and −5 °C (Figure 4, Figures S4–S6). All the protein concentrations were adjusted to 1.5 mM. After a 1-day preservation period at +4 °C (Figure 4a), approximately a 100% survival rate was obtained with the EC solution containing *Tm*AFP or AFP II (denoted *Tm*AFP/EC and AFP II/EC in Figure 4). In contrast, the solution containing AFP I and III yielded only a 60–80% survival rate. With +4 °C preservation for up to 20 days, the survival rate (%) decreased almost linearly for all samples (Figure 4a). Note that the survival rate with EC solution containing BSA was less than 20% after 1 day of preservation; this dropped to 0% with time. The survival rate obtained with *Tm*AFP was always higher than those of the other solutions, namely, rates of 81%, 71%, and 40% of were obtained with *Tm*AFP-dissolved EC solution after 3-, 5- and 10-day preservation periods, respectively. Even after 20 days of preservation at +4 °C, *Tm*AFP yielded a 17% survival rate, while a 0% survival rate was observed with AFP I–III. These results indicate that *Tm*AFP possesses an extremely high cell protection ability at +4 °C.

When the RIN-5F cells were preserved at −2 °C (Figure 4b), the survival rate decreased for all AFP samples compared with +4 °C. For example, the rate decreased to 50% after 5 days of preservation at +4 °C with *Tm*AFP. The survival rates of *Tm*AFP and AFP II were almost identical, i.e., 10% for *Tm*AFP after 20 days of preservation at −2 °C. As shown below, these solutions demonstrated exceptional cell survival at −5 °C; the reason why −2 °C preservation was less effective compared with +4 °C is difficult to understand. Some factors affecting cell metabolism and/or enzymatic function may be unbalanced at −2 °C, although the preservation results with AFPs were much better than those with BSA. The order of cell protection ability of AFPs at +4 °C and −2 °C is as follows: *Tm*AFP > AFP II > AFP III > AFP I.

We next examined the cell preservation effect at −5 °C using the EC solution containing AFP samples (Figure 4c). Note that all such solutions were not frozen and held a supercooling state at −5 °C throughout the preservation experiments. After a 1-day preservation period, the EC solutions containing 1.5 mM of fish AFP I–III or *Tm*AFP yielded a 78% survival rate. The survival rate with AFP I was notably improved, i.e., 50% at +4 °C and −2 °C; however, this increased to 87% at −5 °C. Beyond this period, the survival rates with fish AFP I–III decreased rapidly, i.e., 37%, 68%, and 27% after a 3-day preservation period, respectively (Figure 4c). These rates further decreased linearly to 0% following 20 days of preservation at −5 °C. In contrast, the *Tm*AFP-dissolved EC solution yielded a survival rate of 90%, even after a 5-day preservation period. The rate using *Tm*AFP decreased beyond this period, but was still higher compared with fish AFPs, i.e., 20% of the RIN-5F cells were alive even after 20 days of preservation. In the presence of *Tm*AFP, the cell structure was relatively well-maintained, even after 20 days of preservation at −5 °C (Figure S6). The order of the cell protection ability of AFPs at −5 °C is as follows: *Tm*AFP > AFP II > AFP I > AFP III. The EC solution containing *Tm*AFP gave the best preservation results at any preservation period and temperature with −5 °C-hypothermic preservation.

### 2.4. Further Cell-Viability Improvement by Employing UW Solution

One of the concerns regarding the choice of a cell-preservation solution is that the solution often must not dissolve the AFP sample, which would nullify its function. If the solution contains a protein, it may unexpectedly interact with AFP to make precipitates. We therefore initially selected EC solution, as it consists of only a potassium buffer and sugars, and does not contain any protein [26]. The obtained viability data with the AFP-containing EC solution are therefore attributable to AFP, for which the original antifreeze activity was verified (Figure 1). Based on these results, we next tried the University of Wisconsin (UW) solution that contains many protectants and nutrition, such as cations, phosphates, nucleotides, amino acids, peptides and sugars. Some antioxidants are also present in the UW solution to inhibit oxidization-induced damage to the lipid bilayer (Figure S1). We expected further viability improvements of the RIN-5F cells by employing *Tm*AFP-dissolved UW solution (denoted *Tm*AFP/UW), since the combined use of *Tm*AFP and such beneficial components should be effective for cold-survival. Note that the survival rate of rat hepatocyte was 40% after 7 days of nonfreezing preservation at −4 °C with the UW solution [42]. The polyethylene glycol dissolved in UW solution was also shown to improve the hepatocyte viability from 60 to 90% after 6 days of preservation at −4.4 °C [43]. 

**Figure 5:**
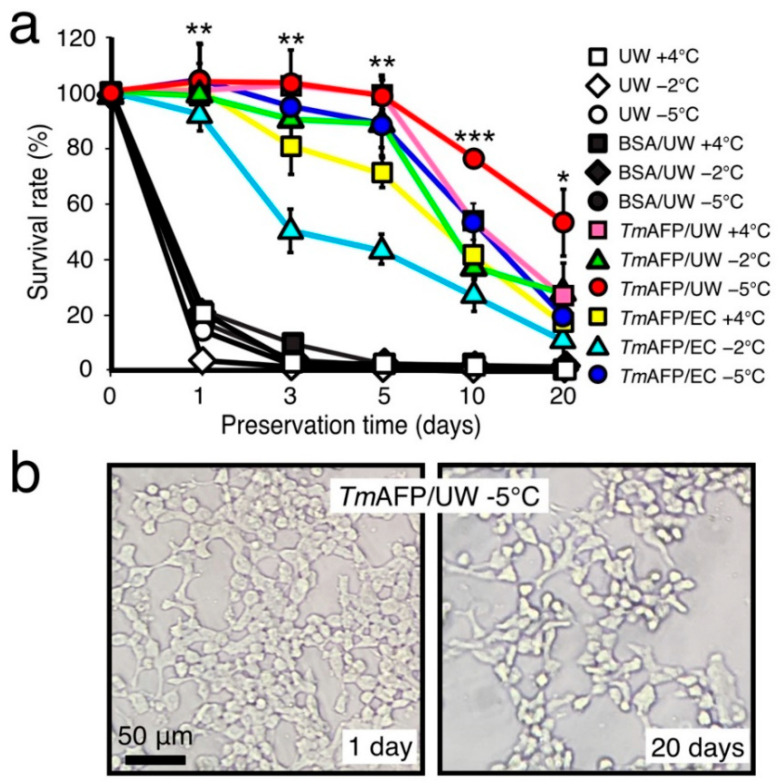
Viability improvement of RIN-5F cells by employing *Tm*AFP-dissolved UW solution. (**a**) Time-dependence of the cell survival rate (%) after nonfreezing hypothermic preservation with UW solution containing 1.5 mM of BSA or *Tm*AFP (denoted like *Tm*AFP/UW). Preservation was performed at +4 °C, −2 °C, or −5 °C for a maximum of 20 days. The *Tm*AFP/UW solution (red circles) achieved a 53% survival rate, even after 20 days of preservation at −5 °C. Asterisk shows that *p*-value vs. control (UW solution) is statistically significant (* *p* < 0.05, ** *p* < 0.01, *** *p* < 0.001). (**b**). Photomicroscope images of the RIN-5F cells after 1- and 20-day preservation periods with the UW solution containing 1.5 mM of *Tm*AFP at −5 °C. The cells are stuck together and elongated in both images, indicating their viability.

Figure 5 compares the time-dependence of the survival rate (%) of RIN-5F cells using the UW solution containing BSA or *Tm*AFP at temperatures of +4 °C, −2 °C, and −5 °C. Note that all solutions were kept in a liquid state throughout of the experiments at both −2 °C and −5 °C. The preservation results with UW solution (UW), BSA/UW solution and the *Tm*AFP/EC solutions shown in Figure 4 are merged into Figure 5a. As shown, the survival rate of RIN-5F cells was 20% after a 1-day preservation period with only UW solution or BSA/UW solution at any temperature. In contrast, *Tm*AFP/UW solution achieved approximately a 100% survival rate after a 5-day preservation period at −5 °C, which did not decrease significantly after 10 days (76%). Consequently, a 53% survival rate was obtained after 20 days of nonfreezing hypothermic preservation at −5 °C when we employed the *Tm*AFP/UW solution (Figure 5a). The RIN-5F cells preserved at −5 °C with *Tm*AFP/UW solution were stuck together and elongated around each other after 20 days of preservation (Figure 5b and Figure S7). The cells after 1 day of preservation were not significantly different in appearance from those after 20 days, although cell counts revealed reduced numbers (Figure 5b). The dramatic improvement in the survival rate (%) with *Tm*AFP/UW solution compared with *Tm*AFP/EC suggests that the former and *Tm*AFP have a synergistic effect on the cells. In other words, *Tm*AFP will function in a −5 °C-chilled, unfrozen UW solution with the help of the protectants and nutrition which are present in that solution.

### 2.5. Mechanistic Consideration for the TmAFP Function

**Figure 6:**
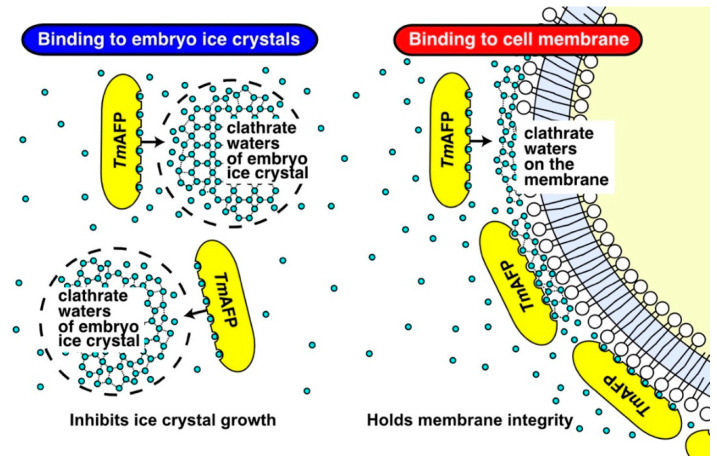
Hypothetical cell protection mechanism of *Tm*AFP during −5 °C nonfreezing preservation. **Left**. *Tm*AFP (yellow ellipse) reveals linearly aligned “ice-like” oxygen atoms on one side of the molecule, which may bind to the tiny seed ice crystals (hatched circles) generated in the −5 °C supercooled UW solution. *Tm*AFP binding inhibits the growth of ice crystals so as not to damage the cells. **Right**. Ice-like oxygen atoms of *Tm*AFP may bind to the clathrate waters located on the lipid bilayer. This mechanism maintained the membrane integrity of cells (right end) in the −5 °C solution. These bindings of *Tm*AFP may ultimately improve the cell viability.

It has been shown that tandem repeats of a 12-residue consensus sequence of *Tm*AFP construct a rounded square pole-shaped structure (Figure 1a), on which an IBS is made of six ladders of the composed sequence -Thr-Cys-Thr- [35,44]. This unique property aligns the sidechain OH-groups of *Tm*AFP at regular intervals, thereby locating ice-like arranged surface waters on this protein (Figure 6). The ice-binding site of fish AFP I–III also contains oxygen atoms that exhibit complementary space matching to the water oxygen atoms in ice crystals, although they are not regularly aligned. 

The regularly arrayed ice-like waters located on the *Tm*AFP are assumed to merge with, and bind to, the latticed waters of the embryo ice crystals generated in the −5 °C supercooled water (Figure 6). *Tm*AFP likely arrests the growth of embryo ice crystals more effectively than fish AFPI–III, as evidenced by higher TH values (4–7 °C) compared with fish AFPs (~1.5 °C). As a consequence, *Tm*AFP likely limits the size of embryo ice crystals in −5 °C-supercooled water, which minimizes physical damage to the cells.

The ability to extend the lifespans of cells at +4 °C has been reported only for fish AFPs [15,16,26,30]. Physicochemical studies have suggested that the adsorption of fish AFPs stabilizes the integrity of lipid bilayer and inhibits ion leakage across the membrane [27,28,29,45]. The AFP III-containing culture medium could indeed support the survival of a bovine embryo for 10 days at 4 °C; this embryo later gave rise to a healthy calf [33]. Hence, one may speculate that *Tm*AFP is also capable of binding to the lipid bilayer under −5 °C hypothermic conditions, and should maintain the membrane integrity to prolong the lifespans of cells. The question of how *Tm*AFP binds to liquid bilayer remains to be answered. It should be noted that some water on the outer surface of the lipid bilayer were regularly proximal to each other and constructed pentagonal clathrates [46,47]. Such ice-like clathrate waters are known to be a key determinant for the “ice-recognition” of *Tm*AFP [48], i.e., the regularly arrayed surface waters located in the IBS of a *Tm*AFP molecule (yellow ellipses in Figure 6) likely undergo complementary binding to clathrate waters on the lipid bilayer, similarly to ice-binding (Figure 6, right), and thereby protecting the membrane integrity in a −5 °C supercooled UW solution.

In conclusion, the present study demonstrates for the first time that insect AFP possesses a hypothermic cell-protection function which is most effective in a −5 °C supercooled solution. Such a function was detected when the AFP was dissolved in the EC solution composed of the buffer detergents, and was enhanced by employing the UW solution containing more protectants. The ice-like waters located on insect AFP are assumed to bind to the waters constructing both embryonic ice crystals and the outer surface of the membrane, which may reduce the chilling damage to cells.

## 3. Materials and Methods

### 3.1. Cell-Preservation Solution

Fish type I–III AFP samples were prepared from the muscle homogenates of barfin plaice (*Liposetta pinnifasciata*), longsnout poacher (*Brachyopsis rostratus*) and notched-fin eelpout (*Zoarces elongatus Kner*), according to established procedures [36,37,38]. The purity check of these samples with 15% SDS electrophoretograms is shown in [49]. *Tm*AFP, purified from the final instar larvae of beetle *Tenebrio molitor*, was provided by the Nichirei Corporation (6-19-20 Tsukiji, Chu-ou-ku, Tokyo 104-8402, Japan), for which we also checked the purity with 15% SDS-PAGE using a minislab electrophoresis kit (AE-6500; ATTO Corp., Tokyo, Japan) (Figure S2). These AFP samples were lyophilized for storage at −30 °C, and were occasionally used with EC solution (KYOWA CritiCare, Japan) or UW solution (Astellas, Japan) to give a final AFP concentration of 0.5–2.5 mM. The EC solution was composed of 99.3 mM KCl, 15.1 mM KH_2_PO_4_, 9 mM K_2_HPO_4_, 10 mM NaHCO_3_ and 194 mM glucose (Osmolarity 355 mM/kg H_2_O, pH 7.4) [29]. The UW solution was composed of 100 mM potassium lactobionate, 50 g/L hydroxyethyl starch, 30 mM raffinose, 5 mM MgSO_4_, 5 mM adenosine, 3 mM glutathione, 1 mM allopurinol and 25 mM KH_2_PO_4_ (pH 7.4). The AFP-dissolved EC or UW solution was cooled to +4 °C overnight and sterilized with a syringe filter (ø = 0.22 µm) (Millex-GP 0.22; Merck Millipore, Burlington, MA, USA) before use.

### 3.2. TH Measurement

The TH value was measured for the AFP samples dissolved in EC solution using a photomicroscope (Leica Microsystems AG, Wetzlar, Germany) equipped with a Linkam 10002L temperature-controlled stage (Linkam Science, London, UK) and a CCD camera, as described previously [18,24]. A 1-µL sample solution was placed in a glass capillary tube (ø = 0.92 mm) (HIRSCHMANN, Eberstadt, Germany). The tube was then set into a house-made copper capillary holder and placed in the photomicroscope stage [49]. The sample was flash frozen at approximately −25 °C to achieve a polycrystalline state of ice crystals. The solution was then warmed to nearly 0 °C to retain a single ice crystal. This ice crystal changed in appearance from a rounded, disk-like shape to a lemon-like morphology or a hexagonal bipyramid when we slightly lowered the stage temperature, indicating AFP-binding [19,50]. For such an ice crystal in the AFP-bound state, we measured the temperature at which the crystal started to melt (*T*_m_). We then secured another lemon- or bipyramidal-shaped, AFP-bound ice crystal in the same solution, the temperature of which was lowered at a rate of −0.1 °C/min. This drop in temperature caused a bursting growth of the ice crystal, which was determined as the nonequilibrium freezing point (*T*_f_). This allowed us to determine the TH value (TH = *T*_m_ − *T*_f_) [21,22]. The measurement was repeated at least three times, and the averaged value is indicated in Figure 1c.

### 3.3. Cell Cultivation

Adherent rat insulinoma cells (RIN-5F, ATCC, CRL-2058) were used as the preservation target in this study. The cells were grown in a flask and incubated at 37 °C in a humid atmosphere containing 5% CO_2_. The cells were then cultured with a RPMI-1640 medium (ATCC modification, Thermo fisher science, Japan), supplemented with heat-inactivated 10% fetal bovine serum (Wako, Japan) [51].

### 3.4. Cell Preservation Experiments

The preservation experiment for the RIN-5F cells was performed according to published procedures [26]. The cells were cultured in a flask to reach an 80%-confluent state, and then detached from the flask with phosphate buffer saline (PBS) containing 0.25% trypsin and 1 mM EDTA (Nacalai tesque, Japan). They were then transferred to a centrifuge tube with 4 mL RPMI-1640 medium. After centrifugation at 3000 rpm for 5 min, the collected cells were resuspended with fresh medium, and their number was counted using a hemocytometer model R1 equipped with a cell counter (Olympus, Japan). The morphology of the cells was also observed with a phase contrast photomicroscope. From this suspension, a small aliquot (100 µL) containing 5 × 10^5^ cells/mL was sucked up and poured into a hollow of the 96-well microplate (BD Falcon, Japan). Following their colonization by 3 days of incubation at 37 °C, we added 0.4% trypan blue (Wako, Japan) dissolved in PBS to stain the dead cells, which allowed us to evaluate the number of living cells before starting the preservation experiment. The RPMI-1640 medium was then replaced with an AFP-containing preservation solution (100 µL). These medium-replaced 96-well plates were put into an incubator at temperatures of +4 °C, −2 °C or −5 °C, in which the RIN-5F cells were preserved for 1, 3, 5, 10 and 20 days (Figure 2a). The living cell number after preservation divided by the number before preservation was defined as the survival rate (%). We used three wells in a 96-well plate on each protein sample and repeated the preservation experiment at least three times. A mean value ± standard deviation was reported for all data. These data were considered statistically significant, as their *p*-value vs. control was less than 0.05 (*p* < 0.05).

## Data Availability

Data is contained within the article or Supplementary materials.

## Supplementary Materials

Supplementary materials can be found at https://www.mdpi.com/article/10.3390/ijms222312680/s1.

## Abbreviations

AFP	Antifreeze protein
*Tm*AFP	*Tenebrio molitor*-derived AFP
BSA	Bovine Serum Albumin
RIN-5F	Rat Insulinoma Cell line code 5F
TH	Thermal hysteresis
IBS	Ice Binding Site
PBS	Phosphate Buffer Saline
EC	Euro Colins
UW	University of Washington
