# Supplementary material for: Subzero Nonfreezing Hypothermia with Insect Antifreeze Protein Dramatically Improves Survival Rate of Mammalian Cells
Source: Int J Mol Sci. 2021 Nov 24;22(23):12680. doi: 10.3390/ijms222312680 (PMC8657916; doi:10.3390/ijms222312680)
Supplement: Supplementary file 1 [file ijms-22-12680-s001.zip › ijms-1454500-supplementary_final.pdf]

Supplementary information for

Subzero non-freezing hypothermia with insect antifreeze protein dramatically improves survival rate of mammalian cells

Akari Yamauchi [1], Ai Miura [2], Hidemasa Kondo [1, 2], Tatsuya Arai [3], Yuji C. Sasaki [3,4], and Sakae Tsuda [1,2.4*]

[1] Graduate School of Life Science, Hokkaido University, Sapporo, 060-0810 Japan.

[2] Bioproduction Research Institute, National Institute of Advanced Industrial Science and Technology (AIST), Sapporo, 062-8517 Japan.

[3] Graduate School of Frontier Sciences, The University of Tokyo, Kashiwa 277-8561, Japan.

[4] OPERANDO Open Innovation Laboratory, National Institute of Advanced Industrial Science and Technology (AIST), Tsukuba, 305-8563 Japan.

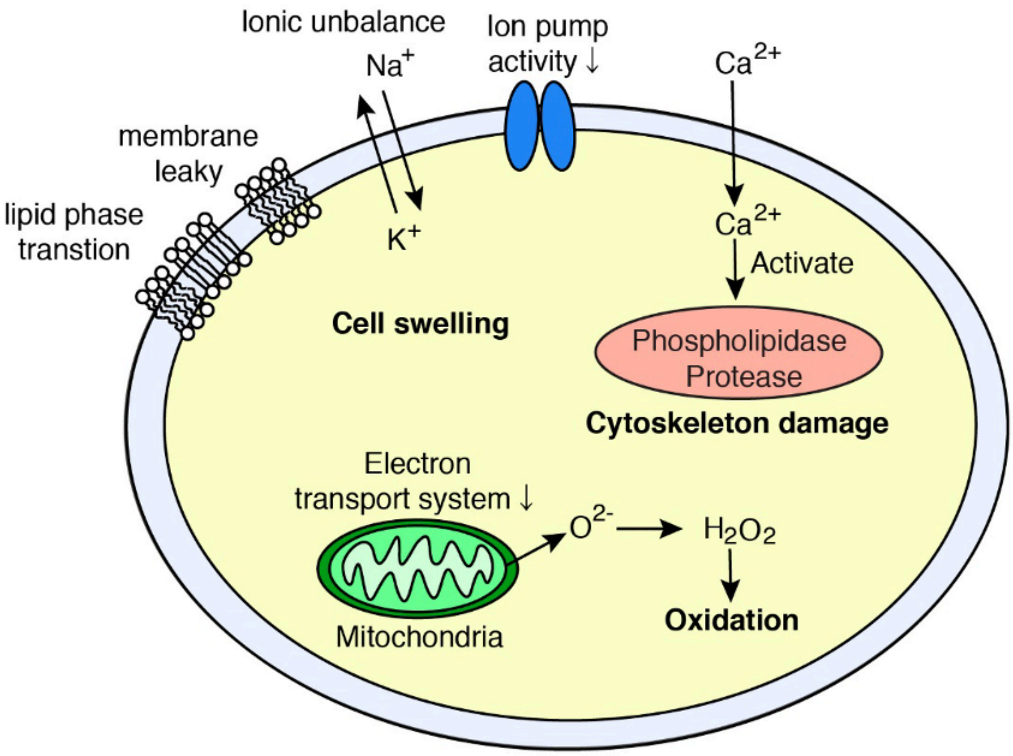

Figure S1. Cold-induced damages on a cell. When mammalian cell is exposed to low temperature, the lipid bilayer undergoes phase transition from liquid crystal to gel state, leading to lower the membrane integrity. The function of membrane proteins relevant to the ionic channels were also affected significantly. These changes make the membrane leaky and modify the osmotic balance in- and outside of the cell. Excessive intracellular calcium especially damages on the cytoskeleton. Mitochondria is also partitioned by lipid bilayer, inner side of which locates the proteins involved in the electron transport system for ATP production. This system is also affected by temperature lowering, thereby accumulating excessive amounts of electrons. Oxygens receive the electrons to become active oxygens, which is converted to peroxygen through hydroxy radicals to oxidize many substances, such as intracellular lipids and proteins. This mechanism is called "cytotoxicity". To secure the membrane integrity will therefore be the primary request in the non-freezing hypothermic cell preservation, for which AFP's protection function is expected (see the references [1, 6 –11] listed in the main text).

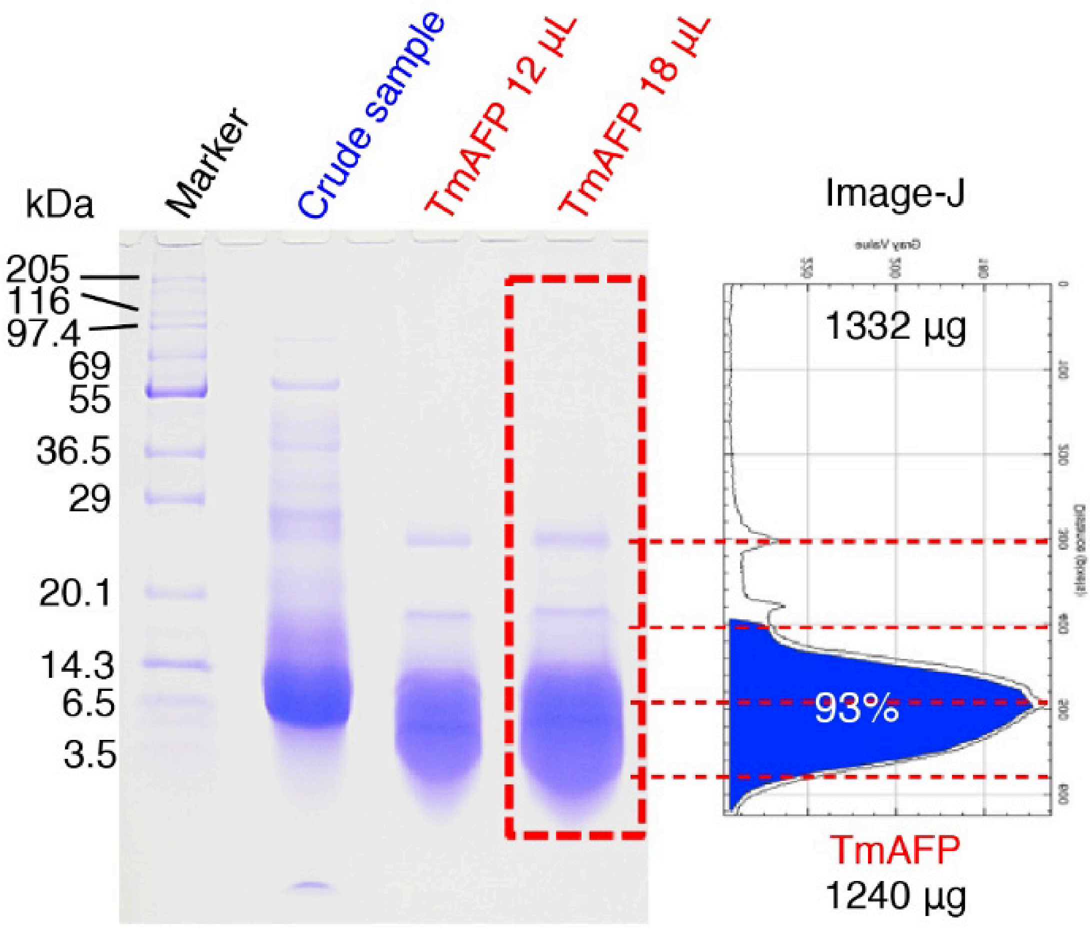

Figure S2. Purity check for the *Tm*AFP sample. Tricine SDS-PAGE (15%) and Image-J (https://imagej.nih.gov/ij/) analysis of the *Tm*AFP samples gift from Nichirei Corporation (6-19-20 Tsukiji, Chuo-ku, Tokyo, 104-8402 JAPAN). The final (3rd) instar larvae of the beetle *Tenebrio molitor* is the resource to obtain both crude and purified native samples of *Tm*AFP. Each sample was dissolved into 25 mM Tris-HCl buffer (pH=7.8) containing 2% SDS and 10% glycerol, which was applied to a 15% precast gel (e-PAGEL E-T15L; ATTO Corporation, Tokyo, JAPAN) set into an electrophoresis tank (AE-6500; ATTO Corporation, Tokyo, JAPAN). The running buffer consists of 25 mM Tris, 25 mM tricine, and 0.05% SDS. The 93% of the sample purity was evaluated based on the electrophoretogram density of *Tm*AFP.

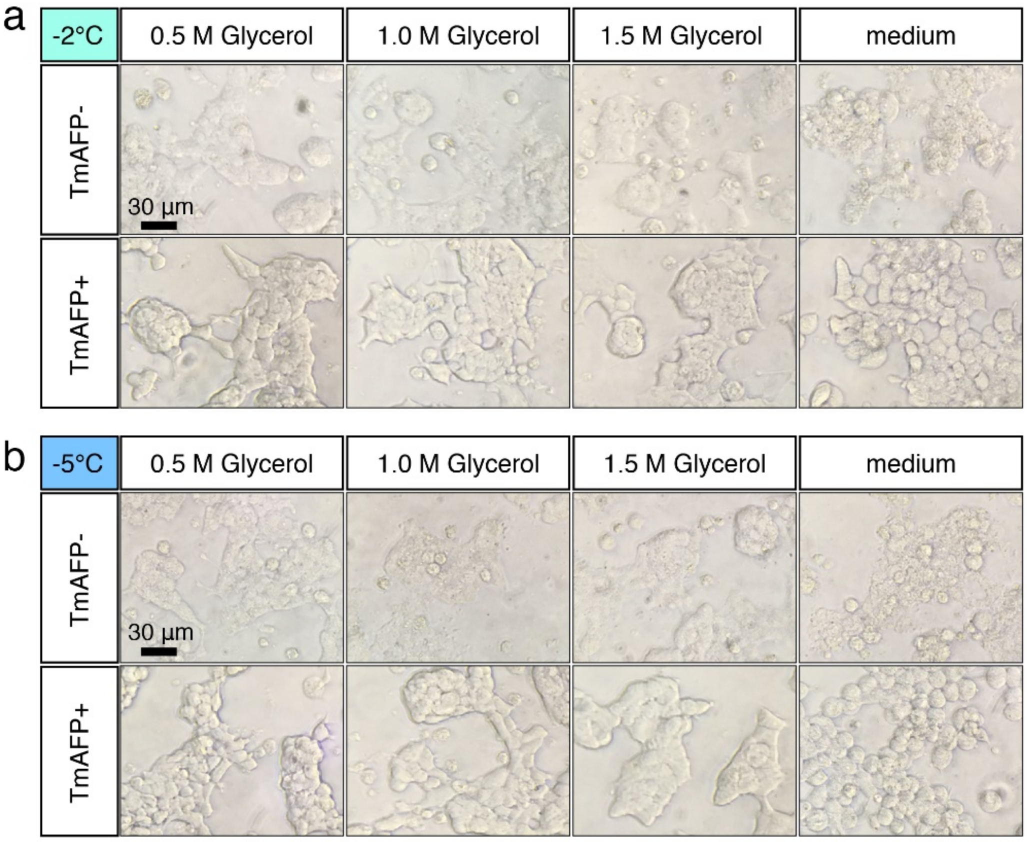

Figure S3. Photomicroscope images of RIN-5F cells after 1-day preservation with glycerol-containing EC solution or RPMI-1640 medium. a. Images of the cells obtained after −2°C-preservation with EC solution plus glycerol or the medium in the absence (–) and presence (+) of *Tm*AFP. **b.** Images obtained after −5°C-preservation with the same set of the solutions. Alive cells are sticked together to be elongated around themselves, while dead cells are distinctly rounded to be collapsed. The latters were clearly recognized by trypan-blue staining, and that their number could be counted with Olympus R1 hemocytometer to evaluate the survival rate (%).

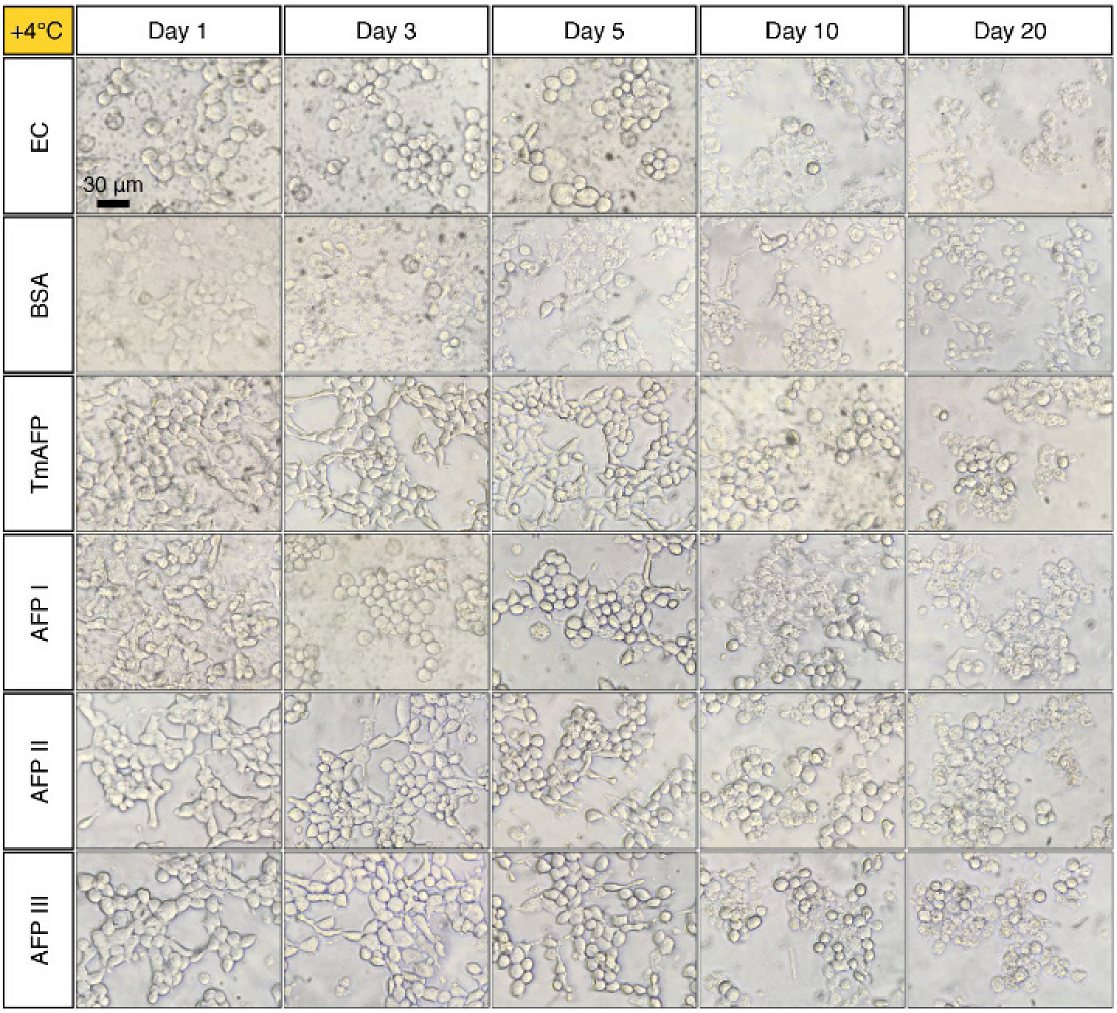

Figure S4. Photomicroscope images of RIN-5F cells after +4°C-preservation for 1−20 days. The images of RIN-5F cells preserved with BSA-, *Tm*AFP- or AFP I–III-containing EC solution for 1, 3, 5, 10 and 20 days at +4°C. The protein concentration was 1.5 mM. Alive cells are sticked together to be elongated around themselves, while dead cells are distinctly rounded to be collapsed.

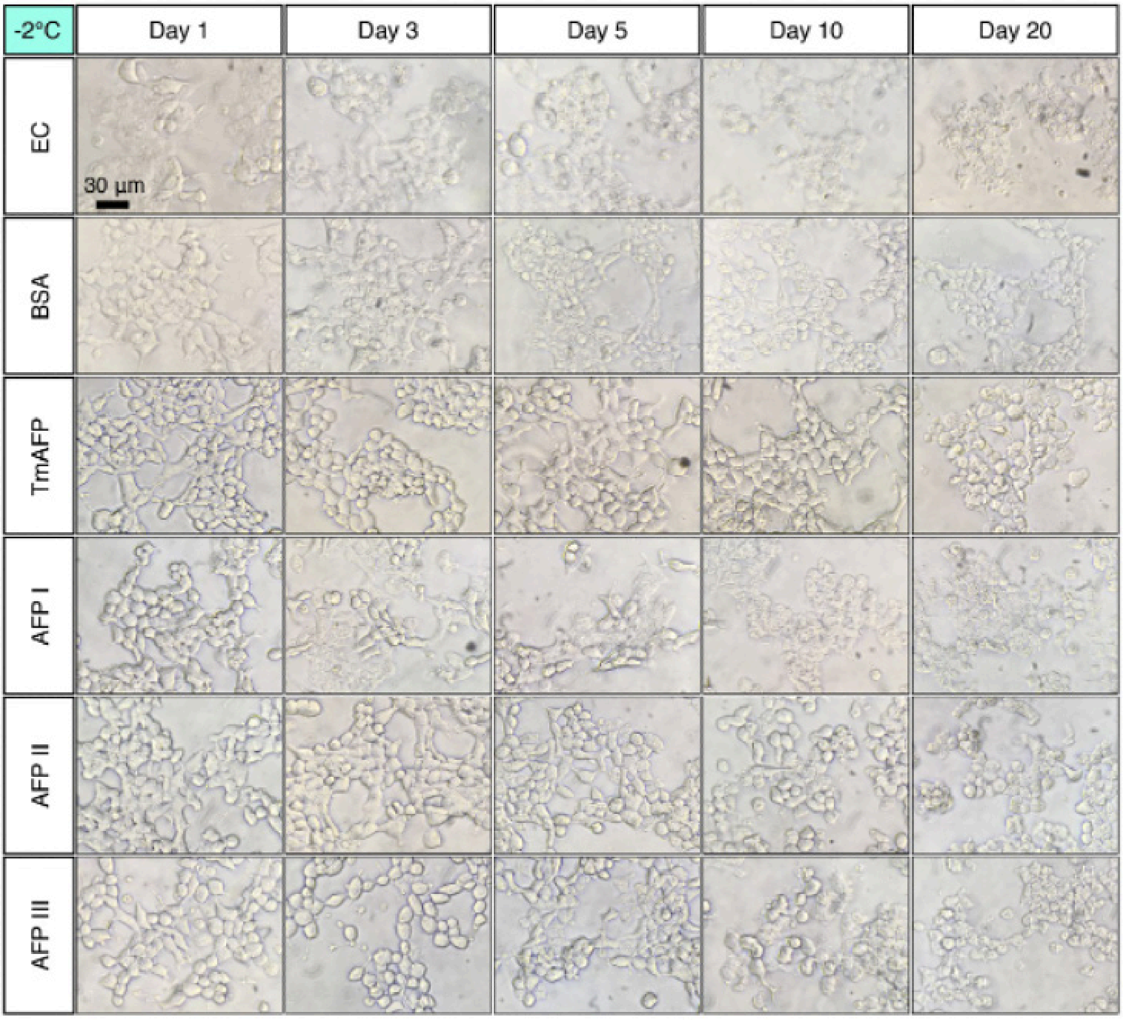

Figure S5. Photomicroscope images of RIN-5F cells after -2°C-preservation for 1−20 days. The images of RIN-5F cells preserved with EC solution containing BSA, *Tm*AFP, or AFP I–III solution for 1, 3, 5, 10 and 20 days at −2°C. Protein concentration was 1.5 mM. Alive cells are sticked together to be elongated around themselves, while dead cells are distinctly rounded to be collapsed.

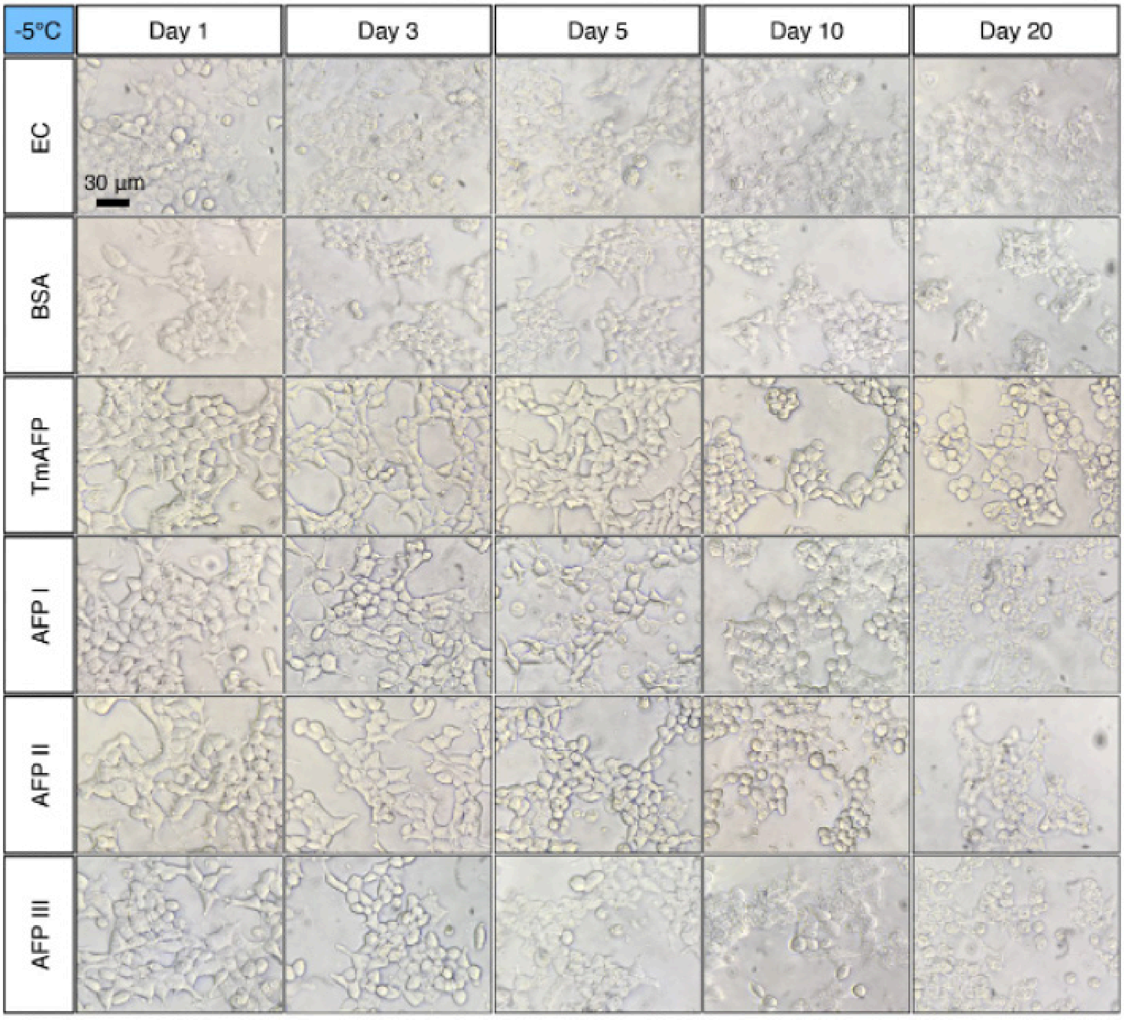

Figure S6. Photomicroscope images of RIN-5F cells after -5°C-preservation for 1−20 days. The images of RIN-5F cells preserved with EC solution containing BSA, *Tm*AFP, or AFP I–III solution for 1, 3, 5, 10 and 20 days at −5°C. Protein concentration was 1.5 mM. Alive cells are sticked together to be elongated around themselves, while dead cells are distinctly rounded to be collapsed.

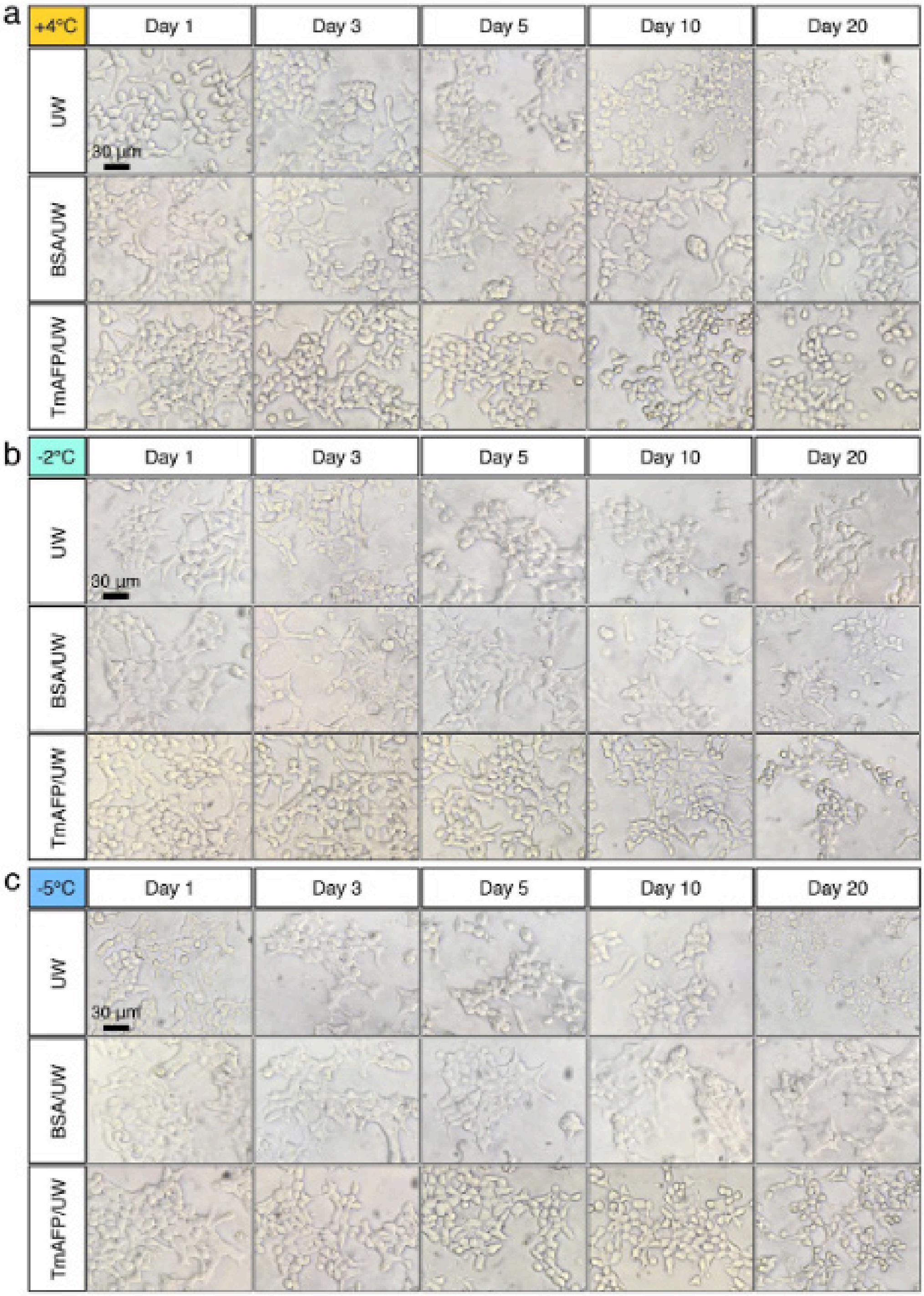

Figure S7. Photomicroscope images of RIN-5F cells preserved with *Tm*AFP-dissolved UW solution at +4°C, −2°C and −5°C. a-c. Images of the cells preserved with UW solution, BSA-dissolved UW solution (BSA/UW) and *Tm*AFP-dissolved UW solution (*Tm*AFP/UW) after +4°C-, −2°C-, and −5°C-preservation for 1, 3, 5, 10 and 20 days. Protein concentration was 1.5 mM.
